# Opportunities for Environmental Noise Mapping in Saudi Arabia: A Case of Traffic Noise Annoyance in an Urban Area in Jeddah City

**DOI:** 10.3390/ijerph13050496

**Published:** 2016-05-13

**Authors:** Mohamed A. Zytoon

**Affiliations:** 1 Department of Industrial Engineering, King Abdulaziz University, P.O. Box 80204, Jeddah 21589, Saudi Arabia; mzytoon@kau.edu.sa; Tel.: +966-12-640-0000 (ext. 72391); 2 Department of Occupational Health & Air Pollution, High Institute of Public Health, Alexandria University, 165 El-Horrya Avenue, Alexandria, Egypt

**Keywords:** environmental noise, traffic noise, noise mapping, public noise annoyance, façade noise levels, grid noise calculation, Jeddah, Saudi Arabia

## Abstract

As the traffic and other environmental noise generating activities are growing in The Kingdom of Saudi Arabia (KSA), adverse health and other impacts are expected to develop. The management of such problem involves many actions, of which noise mapping has been proven to be a helpful approach. The objective of the current study was to test the adequacy of the available data in KSA municipalities for generating urban noise maps and to verify the applicability of available environmental noise mapping and noise annoyance models for KSA. Therefore, noise maps were produced for Al-Fayha District in Jeddah City, KSA using commercially available noise mapping software and applying the French national computation method “NMPB” for traffic noise. Most of the data required for traffic noise prediction and annoyance analysis were available, either in the Municipality GIS department or in other governmental authorities. The predicted noise levels during the three time periods, *i.e.*, daytime, evening, and nighttime, were found higher than the maximum recommended levels established in KSA environmental noise standards. Annoyance analysis revealed that high percentages of the District inhabitants were highly annoyed, depending on the type of planning zone and period of interest. These results reflect the urgent need to consider environmental noise reduction in KSA national plans. The accuracy of the predicted noise levels and the availability of most of the necessary data should encourage further studies on the use of noise mapping as part of noise reduction plans.

## 1. Introduction

The adverse health effects of environmental noise have been extensively studied during the last two decades and evidence of these health effects has accumulated. Sufficient epidemiological studies have found relationships of environmental noise to cardiovascular diseases, annoyance, sleep disturbance, cognitive impairment, and tinnitus [1]. As a result, the importance of environmental noise pollution in shaping urban, environmental, and public health policies throughout the EU and internationally is increasing, albeit at a relatively slow pace [2].

In the EU, the Environmental Noise Directive (END) 2002/49/EC [3] has been established for management of the environmental noise problem, and noise maps have been mandatory for all large agglomerations, major airports, roads, and railways. Noise mapping is simply a means of presenting calculated and/or measured noise levels in a representative manner over a particular geographic area [4]. Since the establishment of the END in 2002, there has been a significant improvement in awareness among the general public and policymakers about the relationship between human exposure to environmental noise and related public health concerns [2].

The END allowed each Member State to use its national method of noise assessment during the first phase of implementation, making it difficult to compare the results from the Member States [4]. For improving the effectiveness of implementing the END, a common approach for assessing noise levels in Europe, namely CNOSSOS-EU, was developed by the European Commission in 2012 [5]. This common assessment method was attached as an annex to the Commission Directive (EU) 2015/996 mandating all Member States to use it from 31 December 2018 onwards [6].

In parallel to the European Commission efforts in environmental noise management, there is an increasing trend in the EU pushing the policy to move beyond the traditional noise control methods towards a more integrated approach of urban sound planning, and some large-scale projects are currently in place [7].

In countries outside the EU, noise mapping has not yet been mandatory. However, some progress has been found in the use of noise maps for environmental noise management. For instance, production of noise maps of several cities all over the world have been mentioned in the literature, such as in the United States [8,9,10], China [11], Hong Kong [12], Korea [13], Brazil [14,15], Argentina [16], and many others.

In KSA, environmental noise, particularly traffic noise, has been found a persistent environmental problem [17,18,19]. This is mainly because KSA has experienced fast development with rapid growth of cities and urban extensions as well as high population growth, which accelerated the demands for increased transportation facilities [18]. For instance, traffic volumes above 300,000 vehicles/day are common on the main roads of Jeddah City [19].

Starting from 2012, the Presidency of Meteorology and Environment (PME) established new Saudi standards of environmental noise, of which community noise standard includes daytime, evening, and nighttime noise levels for three types of area according to area sensitivity [20]. The standard does not mandate city noise mapping, but requires noise modeling and mapping for environmental impact assessment studies. Furthermore, the standard allows using noise prediction methods where direct measurement is not practicable. Nevertheless, the standard is intended to be revised every five years. With increased interest in city (or strategic) noise mapping in KSA, it is likely to be included in a revised version of the standards.

Currently, some research institutions in KSA have shown interest in environmental noise mapping. For instance, King Abdulaziz City for Science and Technology (which is responsible for establishing KSA national research priorities) has recently considered noise mapping of different cities in the Kingdom and noise monitoring and mitigation as part of its research-supporting priority [21]. The growing interest in environmental noise mapping in KSA is shown in some published studies in Al-Dammam City [17] and Riyadh City [21].

In the Al-Dammam noise study [17], direct noise measurement was performed in 122 locations adjacent to main roads and, subsequently, imported into noise mapping software to calculate noise contours. No information was mentioned about other types of data used in the software. On the other hand, the noise map produced for Riyadh City [21] was generated by integrating the data of real equivalent noise (*L*_eqA_) measurements for a period of 40 min (divided between daytime and nighttime) along with GPS data in 5284 locations. These data were imported to ArchGIS9 software (ESRI, Redland, CA, USA) to produce the noise map of the measured acoustic parameter.

It seems that the aforementioned trials of noise mapping in Saudi cities depended mainly on extensive real measurement of noise levels. Due to the complexity, and mainly laborious and expensive task of measuring the acoustic situation of a place, simulations may be used through prediction software informing noise levels in a faster way and less measurement data may be used for calibration of the prediction model [14]. Additionally, noise maps produced from measurement are static, whereas those generated by prediction models are live, allow easy updates, and can separate contributions from all other component noise sources [22].

It is worth mapping environmental noise in KSA metropolitan cities using environmental noise prediction models. These models can be used also in generating public noise annoyance data that are believed to be important in identifying the highly annoyed people who need priority action plans to protect their health. The use of noise mapping and annoyance models requires a large amount of data, e.g., digital maps with GIS data, terrain, buildings geometry, traffic data (road characteristics, traffic flow, speed, transportation type, *etc.*), population data, and others.

The objective of the current study is to test the adequacy of the available data in KSA municipalities for generating urban noise maps and using them for public annoyance analysis, and to verify the applicability of available environmental noise mapping models for KSA. This paper evaluates a model recommended by the END for traffic noise prediction using measurements and noise annoyance surveys in the Al-Fayha District.

## 2. Materials and Methods

### 2.1. Study Area

**Figure 1:**
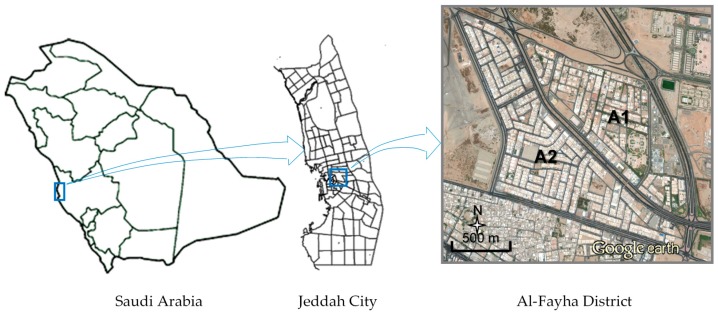
Study area (Al-Fayha District).

**Figure 2:**
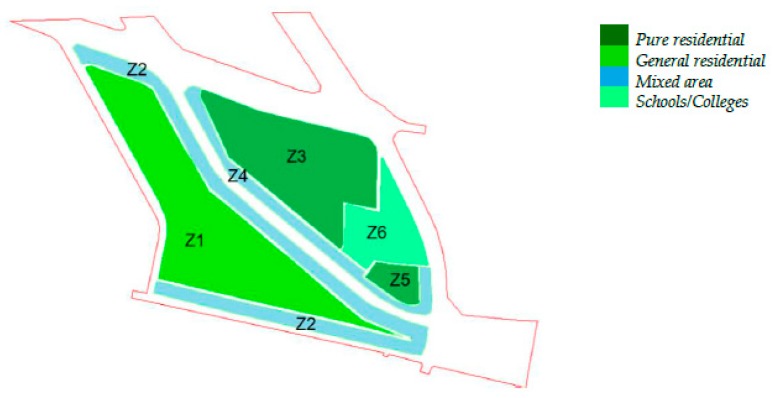
Current planning zones in Al-Fayha District.

The study was conducted in the Al-Fayha District in Jeddah City (Figure 1). The District consisted of two main neighborhoods. The eastern one (A1) was mainly pure residential consisting of groups of detached houses (mostly two-story villas) and some small commercial centers or schools/colleges on the boundaries. The western neighborhood (A2) consisted of general residential multistory buildings (mostly four floors) and mixed residential/commercial buildings on the boundaries. This area had a population density higher than the eastern neighborhood. The current planning zones are shown in Figure 2. The total population of the District was 38,705 inhabitants, distributed over the planning zones as follows: Z1 (71.7%), Z2 (18.7%), Z3 (6.5%), Z4 (2.1%), and Z5 (1.0%). The distribution of inhabitants was calculated based on the Saudi Central Department of Statistics and Information (CDSI) statistics for the average inhabitants per apartment (4.3) and per villa (7.1), and the total number of apartments or villas in each zone. The accuracy of this method was verified by calculating the projected 2015 population of the District based on the available 2010 statistics (number of inhabitants of 33,577 and projected growth rate of 2.9%) which was found consistent with the calculated total number of inhabitants (38,705).

The District was bordered by four streets/roads with moderate to high traffic density and crossed by a road with high traffic density. Therefore, the main contributor to environmental noise in the Al-Fayha District was traffic noise.

### 2.2. Input Data

The following data were obtained from Jeddah Municipality, unless otherwise mentioned:

- Digital maps of Al-Fayha District in the form of GIS files, containing street centerlines, curbs and building boundaries, as well as elements attributes and coordinate system information. Unfortunately, building heights were not available in the GIS file provided by the Municipality. Therefore, both the official urban planning map and the recent aerial photos of the Al-Fayha District had to be reviewed to determine existing buildings’ heights. Also, field survey was conducted to validate the determined buildings’ heights and collect other relevant missing data. For instance, the maps and aerial photos provided by the Municipality were two years old and some of the previously unbuilt land spaces were built at the time of the study into fully constructed buildings. This was considered in the digital map.
- Traffic data for the main roads were obtained from Jeddah Municipality. However, some traffic data were unavailable. Therefore, traffic data for sub-streets as well as missing data of the main roads were obtained by direct measurement. The data obtained were traffic volume, average speed, and vehicle size during the three periods daytime, evening, and nighttime in addition to street surface type and availability of screens.
- The annual average meteorological data were taken from PME annual climate reports of Jeddah.

### 2.3. Data Processing

**Figure 3:**
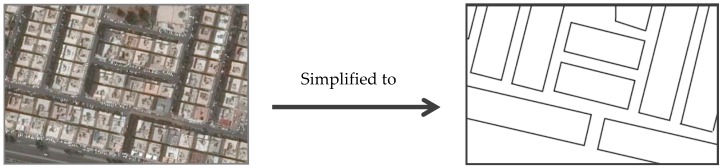
Simplification of the number of buildings: the left figure is a real aerial photo of parcels of multistory houses; the right figure shows treating each parcel as one building.

The data were processed and noise maps were generated using Lima Environmental Noise Mapping Software (B&K Type 7812C). For a complex model, one calculation set may take several days by the software. Some assumptions were considered during data entry for simplification of the model and for shortening calculation time. Using reasonable values of attributes kept the decibel error generally within acceptable limits [23]. The assumptions are summarized as follows:

- Terrain effect was neglected, *i.e.*, the whole district was assumed to be at the same level off the sea since the average slope along the district (from east to west direction) was less than 0.8% (about 0.45°). However, underground tunnels were considered and their contour lines were added to the model.
- Fine details of building geometry were neglected and buildings were smoothed to decrease the number of points and vertices, resulting in a reasonable reduction of model size and faster calculations with minimum error.
- Buildings with the same geometry and height which form parcels of adjacent buildings and having narrow separations among them were considered as one building having one height [16], as shown in Figure 3. Otherwise, numerous data would be collected and entered to the software and too much time would be consumed in calculation.
- For calculation of building height, the first floor height was assumed to be 4 m and the subsequent floors were assumed to be 3 m.
- For all streets and roads, the type of surface was assumed to be the ordinary type (bitumen).

**Figure 4:**
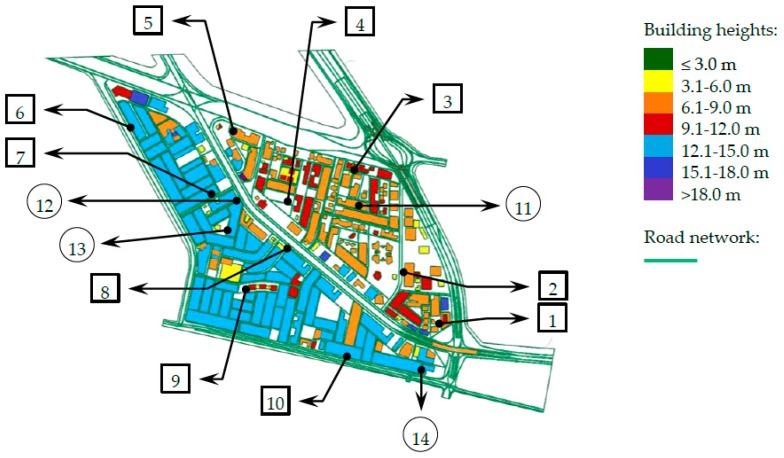
The study model showing building heights and road network, and noise measurement locations in Al-Fayha District (Squares: free field measurements at 1.5 m above ground; Circles: façade measurements).

The study model showing building heights and road network is shown in Figure 4.

### 2.4. Calculation Methods

Calculation methods for road traffic noise consist generally of calculating the level of noise at the source and noise propagation away from the source, however, the details and formulae presented in each method do differ considerably from one method to another [4]. A detailed discussion of the differences between calculation methods is presented elsewhere [4,24]. In this study, since road traffic was the main contributor to noise, the French national computation method “NMPB” that was recommended by END [3] and applied in other studies [16] was used.

The calculated parameters were daytime, evening, and nighttime equivalent continuous A-weighted sound levels. The definitions of the three periods in the Saudi standard of environmental noise are the same as in the END, being: daytime (from 7:00 to 19:00), evening (from 19:00 to 23:00), and nighttime (from 23:00 to 7:00). Calculations were made at vertical height of 1.5 m from the ground level, as recommended by the Saudi standard.

Building façade levels were computed for public annoyance analysis. The calculations were made using the grid point noise levels generated by the NMPB method using a correction of −3 dBA to negate the reflection of the façade before estimating the noise exposure of the inhabitants of the buildings [25]. The day and night levels were calculated at the most exposed façade of dwellings. The most exposed façade was the external wall facing onto and nearest to the specific noise source, as defined in Annex I of END [3]. The revised definition proposed in the CNOSSOS-EU [5] that takes into account the façade with the highest value of *L*_DEN_/*L*_N_ was not applied as the software version used did not support the new definition. The number of inhabitants in each building was calculated using the guidelines of CNOSSOS-EU, where the number of dwellings in each building was multiplied by the average number of inhabitant per dwelling as mentioned in Section 2.1.

Public annoyance was analyzed and noise index was calculated in Lima Environmental Noise Mapping Software using the LärmKennZiffer (LKZ) method (Noise-Evaluation-Index-Method). LKZ method is a linear relationship between noise index (or score) and the exceedance of a prescribed limit value [26,27,28,29]. The noise index was calculated using the following equations (Equations (1) and (2)):

(1) $$\mathrm{Noise Index}=\overset{j}{\underset{i=1}{\sum }}n_{i}\times FC_{i}$$

(2) $$FC_{i}=\left(L_{i}-L_{ref}\right)$$

where *n_i_* was the number of inhabitants in group *i* of a zone consisting of *j* groups that were exposed to different noise levels, *FC_i_* was the façade conflict level (dBA) affecting *n_i_* inhabitants, *L_i_* was the façade noise level (dBA) affecting *n_i_* inhabitants, and *L_ref_* was the reference (or standard) noise value (dBA) for the zone in interest. The Saudi environmental noise standards shown in Table 1 were used as *L_ref_* in annoyance analysis. For illustration, assuming *n* = 50, 200, 100, … inhabitants living in a zone with regulated night noise level *L_ref_* = 50 dBA are exposed to night façade noise levels *L_i_* = 53, 54, 55, … dBA (*i.e.*, façade conflict levels *FC* = 3, 4, 5, …), respectively, then the noise index equals 50 × 3 + 200 × 4 + 100 × 5 + …

The percentage of inhabitants at high risk of annoyance and sleep disturbance were, further, calculated using the percentage of highly annoyed people (%HA) and the percentage of people with high levels of sleep disturbance (%HSD) suggested by Miedema and Oudshoorn [30] and Miedema *et al.* [31], respectively, as follows (Equations (3)–(5)):

(3) $$\%HA=9.994\times 10^{-4}{\left(L_{DN}-42\right)}^{3}-1.523\times 10^{-2}{\left(L_{DN}-42\right)}^{2}+0.538\left(L_{DN}-42\right)$$

(4) $$\%HSD=20.8-1.05L_{N}+0.01486L_{N}^{2}$$

(5) $$L_{DN}=10\times \text{log}\left[\left(15/24\right)\times 10^{L_{D}/10}+\left(9/24\right)\times 10^{\left(L_{N}+10\right)/10}\right]$$

where *L_DN_* was the day-night noise level (dBA), and *L_D_* and *L_N_* were the daytime and nighttime noise levels (dBA), respectively.

### 2.5. Field Measurement and Annoyance Survey

Two sets of measurements were performed. The first set consisted of free field measurements conducted at 10 selected locations for validation of the predicted noise levels at the recommended height in the Saudi noise standards (1.5 m). The selected locations (shown in Figure 4) represented various urban characteristics in both neighborhoods of the District (A1 and A2), *i.e.*, multistory buildings, villas, commercial areas, school areas, areas adjacent to heavy-traffic intersection, and unbuilt open areas. Measurements were made at a vertical level of 1.5 m above ground and 3.5 m far from walls when measuring near buildings. Measurement at each location was performed for three consecutive days. A maximum one weekend day was allowed in one location. However, for various field and time constraints, 4 out of 10 locations did not include weekend measurements, namely locations 1, 2, 6, and 10.

The second set of measurements was conducted to validate the building façade levels at four selected buildings (three buildings in area A2 and one building in area A1), representing pure residential, general residential, and mixed areas (Figure 4). Building 11 was a two-story villa, and façade measurements were taken at two positions (6 m apart) at a height of 5 m. Locations 12 to 14 were multistory houses. Heights of façade measurements were (5 and 8 m), (5 and 8 m), and (8 and 11 m) at buildings 12, 13, and 14, respectively. For achieving consistency with the façade level calculation model, measurements were conducted while the microphone was 0.75–1.0 m in front of the reflecting façade. In this case, a correction factor of −3 dB was used [32] to account for the effect of the reflecting surfaces. For each point, measurement was performed in three consecutive days including two work days and one weekend day (weekend days are Friday and Saturday in Saudi Arabia). Therefore, buildings 11 and 12 were monitored in Wednesday, Thursday, and Friday whereas buildings 13 and 14 were monitored in Saturday, Sunday, and Monday. Using two sound level meters, all measurements were completed in almost two weeks. The number of measurements of the second set was relatively smaller than the first one because façade measurements required house/dwelling owners’ acceptance to keep the equipment inside their residents, using their windows or balconies for connecting the microphone assembly to the sound pressure level meter, and not to operate wall air conditioners during the period of measurement. This, in addition to time limitation of the study, did not allow for getting permission from more residence owners for façade measurements. Nevertheless, the distribution of the four buildings over the three highest populated zones makes them reasonable for verification of the model.

All measurements were conducted using calibrated B&K Type 2250 sound level meter with a free-field microphone type 4189, all enclosed in B&K Portable Noise Monitoring Unit 3655-B. Equipment selection, calibration, and operation was made according to the international standards recommended by the Saudi environmental noise standard [20], *i.e.*, IEC61672-1:2002 for measurement and IEC60942:2003 for calibration.

Annoyance analysis produced by the noise prediction model was verified by a short public noise annoyance survey targeting those whose age was 16 or above [33]. The survey questionnaire was divided into two sections. The first section included questions about demographic data, such as age, sex, marital status, education, floor level (*i.e.*, first, second, *etc.*), and zone number (as given in Figure 2). The second section included questions to measure the level of annoyance during daytime, evening time, nighttime, and overall, as well as sleep disturbance. A five-point Likert scale was used, where the level of annoyance was described as: (1) not at all; (2) slightly; (3) moderately; (4) very; and (5) extremely. The two levels 4 and 5 were used to describe the highly annoyed people [34]. A total of 207 responses were included in the analysis, consisting of 112, 64, and 31 from zones 1, 2, and 3, respectively. The number of responses from other zones was too small to be analyzed (six responses) and, therefore, were excluded.

## 3. Results and Discussion

### 3.1. Data Availability

Table 2 shows the availability of data necessary for noise mapping at the GIS Department of Jeddah Municipality. Except for population and traffic data, all other data needed for noise mapping were available in formats compatible with noise mapping software, such as GIS and CAD files. These file types can be easily imported into the noise mapping software and converted to the proper file type for further processing.

Fortunately, missing data such as number of inhabitant per dwelling are available in other governmental organizations, such as the Central Department of Statistics and Information (CDSI) and Jeddah Urban Observatory (JUO). Also, details of traffic flow in roads and streets can be obtained and regularly updated from Jeddah Traffic Department. In spite of being unavailable in GIS format, building heights (in the form of number of floors) are available from the Municipality as one of the required points of information for issuing building licenses. Furthermore, these data could be verified by using the databases of population statistics, where all demographic characteristics of buildings are collected during the statistical surveys. Through formal collaboration agreements, data exchange between GIS Department in Jeddah Municipality and other governmental organizations, such as CDSI and JUO, can be initiated for the benefit of public health.

### 3.2. Day, Evening, and Night Noise Levels

**Figure 5:**
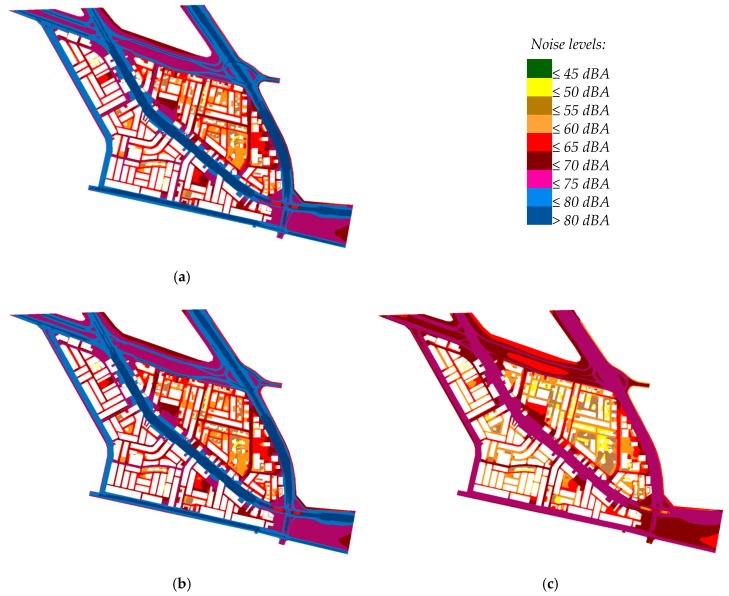
Predicted noise levels due to traffic activities in Al-Fayha District. (**a**) Daytime level (LAeq,D); (**b**) Evening time level (LAeq,E); (**c**) Nighttime level (LAeq,N).

Generally, Figure 5 shows that predicted noise levels were high as compared to the Saudi community noise guidelines. For instance, noise levels higher than 50, 45, and 40 dBA (the Saudi standards for LAeq,D and LAeq,E and LAeq,N, respectively in sensitive areas) were predicted near the premises of schools and mosques in the corresponding period of the day. Furthermore, noise levels in the two areas A1 and A2 mostly exceeded the standards shown in Table 1 during the corresponding periods, except for scattered villas surrounded by wall fences and few multistory buildings that were far from main roads. In agreement with this, Table 3 shows the average façade noise levels of the planning zones in Al-Fayha District, which were higher than the standard levels during daytime and nighttime.

Noise levels from Figure 5 show that, despite the area A1 was a combination of sensitive and mixed area (mainly one or two-story buildings, schools, *etc.*) with lower traffic flow, it was affected by noise levels almost equal to the corresponding levels in the area A2 (multistory buildings, commercial, *etc.*). This might be a combined effect of the lower heights of area A1 buildings, fewer buildings, and more open areas, all resulting in limited barriers to noise propagation, contrary to the area A2 where the buildings might play a role in cutting off the road traffic noises [13]. In addition to these, the very high traffic flow of the surrounding roads from all sides allowed for those high noise levels. The traffic data of the main roads and streets surrounding Al-Fayha District showed very high average daily flow, relative to the population density. These roads received vehicles from/to a large university campus, ring road, and the industrial zone.

Because of the hot climate of Jeddah City, the recreational, social, and leisure activities are normally practiced during the evening period. The traffic flow during this period is high and uniform throughout four hours of the evening. Although the average hourly traffic flow during the evening period is generally higher than that of the daytime period, the predicted LAeq,E values were almost equal or slightly lower than those of LAeq,D. This might be attributed to the sharp reduction of heavy truck flow during the evening period. Some types of these trucks are prohibited by traffic rules and others avoid movement during this peak period. In a recent study carried out by the author in Jeddah (data are not published), it was found that water tanker flow increases community noise levels by up to 1.6 dBA in some residential areas.

The high predicted noise levels during the three periods might be attributed to many reasons. Firstly, high small vehicle flow was observed at the main roads and side streets of Al-Fayha District during daytime, evening, and nighttime. The relatively high traffic flow at side streets resulted from a combination of factors, such as high average passenger vehicle ownership in Jeddah City (about 440 per 1000 persons, as calculated from the official statistics) and the unique pattern of vehicle use in such hot areas where using vehicles for transportation is preferred over walking, even for short distances. Secondly, it was estimated that about 13% of the population in Jeddah depended on water tankers for water supply due to inadequate supply from municipal water network. Furthermore, about 53% of the houses discharged their sewage in septic tanks rather than the municipal sewage network (which does not cover most of the districts). These two factors necessitated the use of water and sewage tanker trucks for the supply and discharge of water, loading the roads/streets excessively with these types of noisy heavy vehicles.

### 3.3. Public Annoyance Analysis

The high noise levels depicted from the produced noise maps necessitated the study of public annoyance due to traffic noise. Table 3 shows that the average conflict levels (difference between average façade noise levels and the corresponding noise standards) were positive, being in the range of 4.6–8.7 dBA during daytime and 7.1–11.2 dBA during nighttime.

**Figure 6:**
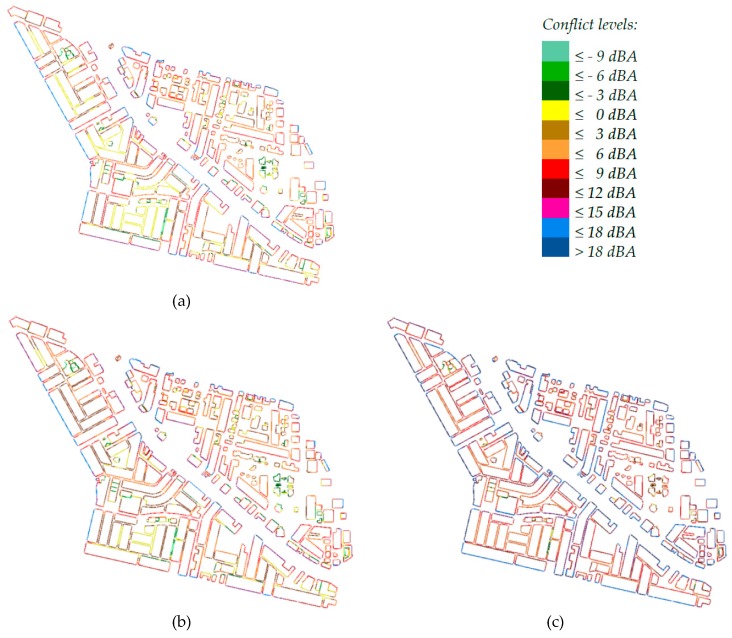
Average Facade noise conflict levels according to Saudi regulation: (**a**) Daytime conflict levels; (**b**) Evening time conflict levels; (**c**) Nighttime conflict levels.

Figure 6 shows the details of average residential façade noise conflict levels during the three periods of the day for all buildings of the District. It is obvious that the conflict levels changed, generally, from day to evening then nighttime in ascending order. Furthermore, conflict levels as high as >15 dBA were found on many buildings, particularly those adjacent to main roads, indicating that people were exposed to severe noise levels. The high conflict levels and percentage of affected population during nighttime relative to other periods necessitates giving priority to the reduction of nighttime traffic noise [13].

Since the planning zones of the area A1 were more sensitive than those in the area A2, their façade noise conflict levels were generally higher. For instance, Table 3 shows that the average façade noise levels during day and night times at the pure residential zone 1 were 57.1 and 52.9 dBA, respectively, and the corresponding daytime and nighttime values at the general residential zone 3 were 61.7 and 57.3 dBA, respectively. However, the average façade conflict values during the same two periods, respectively, were 2.1 and 7.9 dBA at zone 1, and 1.7 and 7.3 dBA at zone 3. This could be explained by two factors. Firstly, the strict standard of the pure residential and educational planning zones included in A1 (zones 3, 5 & 6 in Figure 2) compared to that of the general residential zone of A2 (zone 1 in Figure 2) increased the conflict values at A1, knowing that both areas were adjacent to roads with high traffic density. Secondly, lower floors were exposed to noise levels higher than those at the upper floors. Therefore, the lower building heights at A1 (villas) compared to A2 (multistory) was another reason for higher average façade noise conflict levels. On the other hand, the average conflict levels at the mixed zones 2 (in A2) and 4 (in A1) were close to each other with a difference of 0.2 dBA.

Considering the distribution of population through the District zones, Table 3 shows that 64.5% and 94.9% of the population were affected by noise levels higher than the standard levels of day and night periods, respectively. For instance, 46.7% of the population were exposed to average façade conflict level of 5.2 dBA during daytime whereas 69.4% were exposed to average façade conflict level of 8.9 dBA during nighttime. These numbers reflected the existence of a considerable percentage of highly annoyed persons among the whole populations of the district of interest. Table 3 shows also that the total Noise Index was 145,663 and 326,497 (decibel.person) during the day and night periods, respectively. This index is important in valuing the cost of environmental or community noise in terms of its effect on the annoyed population as well as the cost of mitigation plans. Valuing noise impacts and control measures and balancing their costs is a key challenge in managing the problem from an economic perspective [35].

The LKZ method provides a simple and explainable approach [27], and its dependency on a given standard noise level makes it useful in long-term planning for community noise management. However, it fails to predict the highly annoyed people who should be given the priority in noise action plans. Instead, the %HA and %HSD metrics indicate the probabilities that certain percentages of the population, exposed to certain levels of road traffic noise, would be highly annoyed or have high levels of sleep disturbance, respectively, at a given spot [30,31,36]. Therefore, they were used to predict the burden of disease from environmental noise [1]. In light of this, the two metrics were calculated using the predicted average day and night façade noise levels at the five zones, as shown in Table 3. About one-fifth of Al-Fayha district were estimated to be highly annoyed during daytime and 10.5% were estimated to have high level of sleep disturbance during nighttime. Zones 2 and 4, which are mixed residential/commercial and are adjacent to roads with high traffic flow, had the highest % of annoyance. However, zone 1 had the highest level of annoyance in terms of the number of affected people. For instance, the data of Table 3 can be used to estimate that 52.2% and 54.8% of the total highly annoyed and highly sleep disturbed people of the district, respectively, belonged to zone 1.

As mentioned earlier, a traffic noise annoyance survey was conducted to test the validation of the predicted annoyance levels. Table 4 presents a summary of the sampled population characteristics as well as the results of public annoyance. Only results from zones 1, 2, and 3 are shown as the responses from other zones were too limited to be included. Obviously, all traffic noise annoyance metrics were highest in zone 2, followed by zone 1, and finally zone 3. This is in agreement with the order of average façade levels of the same zones during day and night (Table 3).

The predicted %HA and %HSD were compared to the survey %HA (as %highly annoyed day-night in Table 4) and survey %HSD (as %highly sleep disturbed in Table 4). The comparison is presented in Table 5 which shows that there was a good agreement between the predicted and survey high annoyance metrics. The differences were statistically insignificant at the 0.05 level as shown by the Z-test for two independent proportions. The survey results of zone 2 were relatively higher than the predicted ones, which might be attributed to a relatively high percentage of those living in the most crowded eastern section of zone 2 in the sample. Their higher perception of traffic noise lifted up the %HA and %HSD. On the other hand, the lower perception of noise annoyance by zone 3 inhabitants as compared to the prediction could be due to better acoustical insulation of their houses. For instance, most zone 3 houses were provided with additional screens above the guard walls of their houses for more privacy, which resulted in noise reduction as well.

### 3.4. Comparison of Saudi and END Grid Calculation

The new Saudi noise standards recommend measuring community noise at a height between 1.2 and 1.5 m above ground [20], whereas the recommended calculation height for noise mapping in END is 4 m above ground [25]. In this study, the calculation of traffic noise was made at the two heights 1.5 and 4 m. The results from the two calculation heights were compared to each other in terms of affected inhabitants at various façade noise levels during daytime and nighttime. Table 6 shows that the % inhabitants affected by façade noise levels higher than the standards were higher when using the END recommended height of 4 m, which means that the calculation height recommended by the Saudi standard was relatively less conservative. However, the differences in % inhabitants affected by exceeding levels between the two heights were slight with maximum values of 1.1% and 0.9% for daytime and nighttime periods, respectively. In spite of being slight, these differences could be reflected in the form of savings in the reduction of noise levels.

Annoyance analysis was made by interpolating grid calculation results in this study. It was found in another study that grid interpolation resulted in higher percentage of annoyed population as compared to direct façade calculation [37]. Therefore, analysis of annoyance using grid calculations based on the height above ground recommended by the Saudi standard might give more accurate façade noise levels than that of the height recommended by the END.

### 3.5. Comparison of the Predicted and Measured Noise Levels

Table 7 shows the measured noise levels (locations 1–10) and façade conflict levels (locations 11–14) compared to the corresponding levels predicted by the model. The ranges of the difference between the measured and the predicted noise levels at the selected locations (1.5 m free-field levels) were from −3.3 to 0.2, from −2.7 to 0.5, and from −3.1 to 0.7 dB for day, evening, and nighttime, respectively. On the other hand, the difference ranges of façade conflict levels at the last four locations were −2.9 to 0.1, −1.9 to 2.1, and −4.5 to 2.6 dBA for day, evening, and nighttime, respectively. These deviations could be acceptable in comparison to deviations found in other noise mapping applications [16].

**Figure 7:**
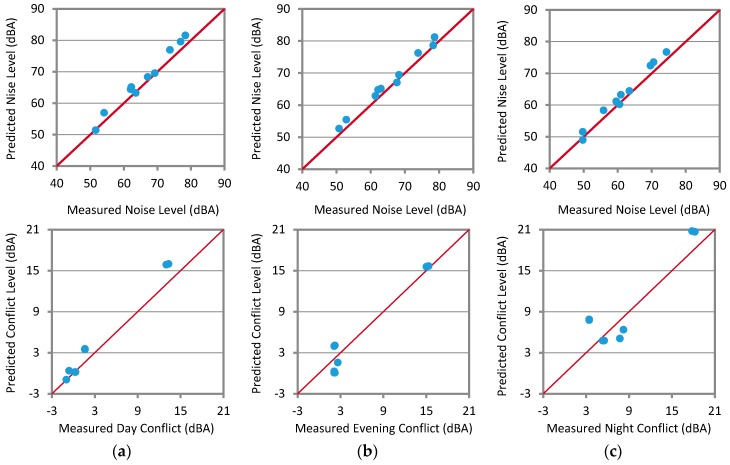
Predicted *vs.* measured noise levels (**upper** figures) and façade conflict levels (**lower** figures). Points: actual correlation; Straight lines: 100% agreement. (**a**) Daytime levels; (**b**) Evening time levels; (**c**) Nighttime levels.

Figure 7 shows the strong agreement between the predicted and the measured noise levels in this study. This good agreement was obtained despite the dynamic error was set to be 1.0 dBA in the prediction model and some settings were modified to accelerate the calculation on the debt of accuracy. It is, therefore, expected that if these settings were modified for maximum accuracy, the average error (variation) of the predicted noise levels from the measured values would, most likely, be lower. However, the calculation time would be much longer.

It should be kept in mind that there were many sources of uncertainty in noise mapping, such as model inputs, uncertainty propagation, model structure, and evaluation data [25,38]. Similar to the study of Ausejo *et al.* [16], the higher uncertainty source was due to road traffic flow (uncertainty group B: 0.5–1.0 dBA) and speed (uncertainty group C: 1–3 dBA) for both light and heavy vehicles (heavy vehicle flow <30% of the overall flow). This resulted in overall uncertainty of about 3.0 dBA in noise predictions of day, evening, and night noise levels. Traffic flow data in this study were collected in a way classified as moderate in terms of cost as proposed by WG-AEN [25], resulting in uncertainty of about 3.0 dBA.

**Figure 8:**
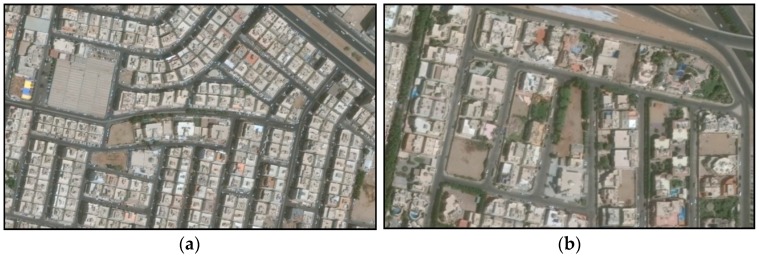
Aerial photos of the two types of houses in Al-Fayha district: (**a**) multistory houses; (**b**) Detached houses (Villas).

The same uncertainty sources apply also for façade noise level prediction. However, since the calculations of façade noise levels are based on 3D models, building geometry could be an important source of uncertainty. In façade noise level calculations, the majority of buildings were modeled as cubes by eliminating actual façade details for simplification of data processing and reducing calculation time. The effect of this can be illustrated using the data of façade conflict levels in Table 7, which shows that the mean difference between the measured and predicted façade conflict levels at location 11 was high and negative in all cases (from −1.9 to −4.5 dBA) compared to locations 12, 13, and 13. The buildings in the later three locations were regular multistory houses (consisting of almost similar apartments), as shown in Figure 8a. On the other hand, location 11 represented zone 3 which consisted, mostly, of detached houses (villas) having irregular structures as shown in Figure 8b. Façade level real measurements showed that, besides the above-mentioned uncertainty sources, simplification of the complex geometry of zone 3 houses further increased the predicted levels above the real values. 

Another potential source of uncertainty associated with the model simplification was the canyon effect resulting from simplification of a parcel of adjacent similar buildings into one building, as shown in Figure 3. This affect the predicted traffic noise propagation by increasing the surface area of parallel building facades which cause multiple sound reflections as well as sound scattering within the urban canyon [39,40]. The result of this was, probably, increased noise levels.

In addition to the uncertainty associated with the calculation model, there were many uncertainty sources during measurement such as errors from measurement chain (e.g., sound level meter), source, meteorological conditions, measurement location, and residual sound [32,41,42]. The combined uncertainties of measurement of the 14 locations were calculated and converted to the expanded uncertainty using a coverage factor of 2 to obtain a 95% confidence level [16,32,41]. The expanded uncertainty of the 10 free-field 1.5 m noise level measurements ranged between 1.6 and 2.0 dBA for the three periods, as shown in Table 7. On the other hand, the measurement expanded uncertainty of the four façade noise levels were higher, being in the range 1.8–2.5 dBA. This was a result of the added uncertainty of the microphone position in façade noise measurements.

## 4. Conclusions

In this study, traffic noise levels were predicted and public annoyance analysis was performed in Al-Fayha District, Jeddah City, Saudi Arabia using noise mapping approach. The predicted noise levels during the three time periods, *i.e.*, daytime, evening and nighttime, were found higher than the maximum recommended levels established in the Saudi environmental noise standards. Annoyance analysis revealed that high percentages of the District inhabitants were highly annoyed, depending on the type of planning zone and period of interest. These results should be an alarm that the environmental noise, in particular traffic noise, might constitute a serious public health problem in the large cities of Saudi Arabia. For controlling this problem, environmental noise reduction plans should be in place in large cities. The first step in these plans is the assessment of the magnitude of the problem through noise mapping and public noise annoyance analysis. In this study, prediction of traffic noise using noise mapping in a Saudi environment was proven to be in agreement with the real field measurements. Furthermore, the results from annoyance analysis using the percentage of highly annoyed people approach, in particular, were very close to the results obtained from a field survey of traffic noise annoyance in the same District. This should encourage the application of noise prediction and mapping models in all large cities as a main element in environmental noise management plans to overcome the high cost of field measurement, which is the most commonly used approach in Saudi Arabia so far. However, noise mapping requires the availability of various types of datasets and supporting tools. The current study revealed that the infrastructure required for the application of noise mapping in large Saudi cities is almost provided in city municipalities, such as updated GIS data and maps, and proper noise map publishing platforms that are accessible to the public. For instance, Jeddah Municipality established an attractive and highly accessed (by the public) web application called Jeddah Geographical Explorer, which presents a lot of information related to the City in the form of layers on a map of Jeddah. Noise maps and annoyance data of Jeddah districts can be presented to the public through this application for raising their awareness of the problem. On the other hand, some other data, such as traffic and population data are available at governmental authorities other than the municipalities, and they can be easily accessible to the municipalities through collaboration within the context of a national environmental noise reduction plan.

The current study aimed at studying the potential for noise mapping and noise annoyance analysis in Saudi Arabia through application in one district in Jeddah City. However, further research is necessary to cover larger urban areas and the whole City as well. This will allow consideration of more types of input data, such as complex topography forms. In addition, further studies are recommended on the various options available to the decision makers of large cities in Saudi Arabia for management of the environmental noise problem. It is also recommended that public awareness and perception of environmental noise annoyance be studied in more details to include larger samples among Saudi residents.

## Figures and Tables

**Table 1:** Saudi standards for community noise.

Category of Residential Area	LAeq, D (dBA) (7:00–19:00)	LAeq, E (dBA) (19:00–23:00)	LAeq, N (dBA) (23:00–7:00)
Sensitive (hospitals, schools, worship, touristic, *etc.*)	50	45	40
Mixed (sparse population, suburban, hotels, hostels, *etc.*)	55	50	45
Non-sensitive (mixed residential and commercial, retail, financial, *etc.*)	60	55	50

**Table 2:** Availability of data necessary for noise mapping at the GIS Department of Jeddah Municipality.

Type of Data	Availability in Jeddah Municipality	Availability Elsewhere
Digital maps	Available in GIS format (shape files), including all sub-municipalities and district boundaries, street centerlines, curb lines, land parcels, and buildings.	-
Local urban planning maps	Available in both digital and hard format showing residential, commercial, governmental, services, green areas, historical and industrial zoning plans of all districts. Type of residence includes villas (one or two-story), multi-story buildings, and old residences.	-
Terrain characteristics	Available in GIS format, including natural and man-made topographical properties.	-
Building information	Available in GIS format, but does not include heights (number of floors) or number of inhabitants.	Building heights are found in buildings‘ license data but not imported to the GIS digital data.
Population information	Only population distribution over districts is available. No information is available about number of inhabitants per dwelling.	Available from the databases of population statistics of the CDSI.
Traffic data	Only traffic light locations are available in GIS format. Information about traffic flow is limited and sparse in hard format.	A current project for automatic traffic flow counting in main roads and crossings; Recent research studies; Environmental impact assessments for new projects.
Roads characteristics	Type of pavement, direction of flow, utility lines (electricity, telephone, water, sewerage, *etc.*), manholes, lights, trees, *etc.* are available as GIS files.	-
Aerial photographs	2013 aerial photos are available.	-
Meteorological data		PME statistics.
Continuous update	Last update of GIS files used in this study is March 2014.	-

**Table 3:** Average noise annoyance statistics due to excess noise levels (LKZ method) and highly annoyed population at Al-Fayha District.

Zone Number (See Figure 2)		Z1	Z2	Z3	Z4	Z5	Totals
**Zone day/night standard levels (dBA)**		60/50	60/50	55/45	60/50	55/45	
**Zone population**		27,750	7245	2523	816	368	**38,705**
**Daytime statistics**	Average façade noise level (dBA)	64.2	71.6	59.6	72.7	59.8	
	Average façade conflict ^1^ (dBA)	5.2	7.6	4.6	8.7	4.8	
	Affected population	18,057	4391	1681	523	301	**24,953**
	% from zone population ^2^	65.1	60.6	78.5	64.1	81.8	
	% from total District population ^3^	46.7	11.3	4.3	1.4	0.8	**64.5%**
	Noise index ^4^	97,231	34,748	7654	4516	1514	**145,663**
**Nighttime statistics**	Average façade noise level (dBA)	57.9	63.5	52.1	65.2	53.3	
	Average façade conflict ^1^ (dBA)	8.9	9.5	7.1	11.2	8.3	
	Affected population	26,873	6736	2132	668	354	**36,763**
	% from zone population ^2^	96.8	93	84.5	81.9	96.2	
	% from total District population ^3^	69.4	17.4	5.5	1.7	0.9	**94.9%**
	Noise index ^4^	236,559	63,444	15,662	7396	3436	**326,497**
	%HA ^5^	17.9	30.4	11.3	33.9	12.1	**20.1**
	%HSD ^6^	9.8	14.0	6.4	15.5	7.1	**10.5**

Notes: ^1^ Calculated using Equation (2); ^2^ % from zone population = (Affected population in a given zone ÷ Total population of the same zone) × 100; ^3^ % from total population = (Affected population in a given zone ÷ Total population of the District) × 100; ^4^ Calculated using Equation (1); ^5^ Calculated using Equation (3); ^6^ Calculated using Equation (4).

**Table 4:** Summary of the sampled residents' characteristics and traffic noise annoyance survey results.

Measured Factor	Zone 1 (112 Responses)	Zone 2 (64 Responses)	Zone 3 (31 Responses)
Sample mean age (± S.D.)	30.4 (± 9.2)	31.5 (± 13.4)	32.7 (± 14.3)
Mean floor level (± S.D.)	2.1 (± 0.6)	2.4 (± 0.5)	1.7 (± 0.6)
% Male	67.9%	75.0%	71.0%
% Married	39.3%	56.3%	64.5%
Mean score of daytime annoyance (± S.D.)	2.1 (± 1.3)	2.8 (± 1.4)	1.9 (± 1.0)
% Highly annoyed during daytime ^1^ (95% C.I.)	17.9% (11.3, 26.2)	31.3% (20.2, 44.1)	12.9% (3.6, 29.8)
% Annoyed to any level during daytime ^2^ (95% C.I.)	57.1% (47.4, 66.5)	75.0% (62.6, 85.0)	54.8% (36.0, 72.7)
Mean score of evening annoyance (± S.D.)	2.2 (± 1.2)	2.9 (± 1.4)	1.8 (± 1.0)
% Highly annoyed during evening ^1^ (95% C.I.)	14.3% (8.4, 22.2)	31.3% (20.2, 44.1)	9.7% (2.0, 25.8)
% Annoyed to any level during evening ^2^ (95% C.I.)	67.9% (58.4, 76.4)	75.0% (62.6, 85.0)	54.8% (36.0, 72.7)
Mean score of nighttime annoyance (± S.D.)	2.0 (± 1.0)	2.7 (± 1.4)	1.7 (± 0.9)
% Highly annoyed during nighttime ^1^ (95% C.I.)	10.7% (5.7, 18.0)	25.0% (15.0, 37.4)	9.7% (2.0, 25.8)
% Annoyed to any level during nighttime ^2^ (95% C.I.)	64.3% (54.7, 731)	75.0% (62.6, 85.0)	51.6% (33.1, 69.8)
Mean score of sleep disturbance (± S.D.)	1.6 (± 1.0)	2.3 (± 1.0)	1.4 (± 0.8)
% Highly sleep disturbed ^1^ (95% C.I.)	8.0% (3.7, 14.7)	18.8% (10.1, 30.5)	3.2% (0.1, 16.7)
% Sleep disturbed to any level ^2^ (95% C.I.)	33.9% (25.3, 43.5)	62.5% (49.5, 74.3)	25.8% (11.9, 44.6)
Mean score of day-night annoyance (± S.D.)	2.1 (± 1.1)	2.8 (± 1.3)	1.8 (± 0.9)
% Highly annoyed day-night ^1^ (95% C.I.)	18.8% (12.0, 27.2)	37.5% (25.7, 50.5)	9.7% (2.0, 25.8)
% Annoyed to any level day-night ^2^ (95% C.I.)	71.4% (62.1, 79.6)	81.3% (69.5, 89.9)	71.0% (52.0, 85.8)

Notes: ^1^ Score 4 or 5; ^2^ Score >1.

**Table 5:** Comparison between predicted and survey %HA and %HSD in three residential zones.

Zone	%HA			%HSD		
	Predicted	Survey	*p* ^1^	Predicted	Survey	*p* ^1^
Z1	17.9	18.8	0.818	9.8	8.0	0.493
Z2	30.4	37.5	0.242	14.0	18.8	0.332
Z3	11.3	9.7	0.762	6.4	3.2	0.326

Note: ^1^ *p*-value for testing the difference between the two percentages (predicted and survey) at the 0.05 significance level.

**Table 6:** Distribution of inhabitants in relation to day and night façade noise levels: comparing results of calculation based on Saudi and END recommended grid calculations height.

Façade Noise Level (dBA)	% Affected Inhabitants during Daytime			% Affected Inhabitants during Nighttime		
	KSA Grid Calculations Height (1.5 m)	END Grid Calculations Height (4 m)	Difference (END—KSA)	KSA Grid Calculations Height (1.5 m)	END Grid Calculations Height (4 m)	DIFFERENCE (END—KSA)
≤45	0.2	0.1	−0.1	2.6	1.6	−1.0
>45–50	2.4	1.5	−0.9	3.9	3.2	−0.7
>50–55	4.0	3.5	−0.5	28.6	28.9	0.3
>55–60	36.3	35.8	−0.4	34.6	34.3	−0.2
>60–65	26.8	26.9	0.2	15.3	16.2	0.9
>65–70	15.3	16.4	1.1	8.4	8.6	0.2
>70–75	9.1	9.3	0.3	6.6	7.1	0.5
>75	6.0	6.4	0.4	0.02	0.03	0.01

**Table 7:** Comparison of predicted and measured noise levels.

Measurement Location (Area & Zone Numbers)	Daytime Noise or Façade Conflict Levels (dBA)			Evening Noise or Façade Conflict Levels (dBA)			Nighttime Noise or Façade Conflict Levels (dBA)			Expanded Uncertainty (dBA)	
	Meas.	Pred.	Diff. ^1^	Meas.	Pred.	Diff.	Meas.	Pred.	Diff.	Meas.	Pred.
Free-field 1.5 m noise levels:											
1 (A1, Z5) ^2^	62.2	65.2	−3.0	62.9	65.2	−2.3	60.5	60.3	0.2	±1.8	±3.0
2 (A1, Z6) ^2^	54.1	57.0	−2.9	52.8	55.5	−2.7	49.7	51.6	−1.9	±1.9	±3.0
3 (A1, Z3) ^3^	63.5	63.3	0.2	61.3	62.9	−1.6	59.5	61.2	−1.7	±1.8	±3.0
4 (A1, Z3) ^3^	69.2	69.6	−0.4	68.2	69.5	−1.3	63.4	64.5	−1.1	±1.6	±3.0
5 (A1, Z4) ^4^	62.0	64.5	−2.5	62.1	64.8	−2.7	55.8	58.4	−2.6	±1.9	±3.0
6 (A2, Z1) ^2^	73.7	77.0	−3.3	73.8	76.3	−2.5	69.6	72.5	−2.9	±1.9	±3.0
7 (A2, Z1) ^3^	67.1	68.4	−1.3	67.6	67.1	0.5	60.9	63.3	−2.4	±1.9	±3.0
8 (A2, Z2) ^4^	78.3	81.6	−3.3	78.6	81.3	−2.7	74.3	76.8	−2.5	±1.9	±3.0
9 (A2, Z1) ^4^	51.6	51.5	0.1	50.7	52.7	−2.0	49.7	49.0	0.7	±2.0	±3.0
10 (A2, Z2) ^2^	76.9	79.6	−2.7	78.2	78.7	−0.5	70.5	73.6	−3.1	±2.0	±3.0
Façade noise conflict levels at various heights:											
11 (A1, Z3) ^4^, height: 5 m	1.6	3.5	−1.9	2.1	4.0	−1.9	3.4	7.8	−4.4	±1.8	±3.0
height: 5 m	1.6	3.6	−2.0	2.2	4.1	−1.9	3.4	7.9	−4.5	±1.8	±3.0
12 (A2, Z2) ^4^, height: 5 m	13.3	16.0	−2.7	15.3	15.7	−0.4	17.8	20.8	−3.0	±2.5	±3.0
height: 8 m	13.0	15.9	−2.9	15.0	15.6	−0.6	18.2	20.7	−2.5	±2.5	±3.0
13 (A2, Z1) ^3^, height: 5 m	−0.6	0.4	−1.0	2.6	1.6	1.0	8.2	6.4	1.8	±1.9	±3.0
height: 8 m	−1.0	−0.9	−0.1	2.1	0.3	1.8	7.7	5.1	2.6	±1.9	±3.0
14 (A2, Z2) ^3^, height: 8 m	0.3	0.2	0.1	2.2	0.1	2.1	5.5	4.8	0.7	±1.8	±3.0
height: 11 m	0.2	0.2	0.0	2.1	0.1	2.0	5.3	4.8	0.5	±1.8	±3.0

Notes: ^1^ Difference (Diff.) = Measured (Meas.) − Predicted (Pred.); ^2^ Measurements did not include weekend days; ^3^ Measurements were during Saturday, Sunday and Monday; ^4^ Measurements were during Wednesday, Thursday, and Friday.
